# Piwi-interacting RNAs as novel prognostic markers in clear cell renal cell carcinomas

**DOI:** 10.1186/s13046-015-0180-3

**Published:** 2015-06-14

**Authors:** Jonas Busch, Bernhard Ralla, Monika Jung, Zofia Wotschofsky, Elena Trujillo-Arribas, Philipp Schwabe, Ergin Kilic, Annika Fendler, Klaus Jung

**Affiliations:** Department of Urology, Research Division, University Hospital Charité, Schumannstr. 20/12, 10117 Berlin, Germany; Berlin Institute for Urologic Research, Berlin, Germany; Laboratorio clinico, Hospital Universitario Virgen Del Rocio, Sevilla, Spain; Center for Musculoskeletal Surgery, University Hospital Charité, Berlin, Germany; Institute of Pathology, University Hospital Charité, Berlin, Germany; Department of Signal Transduction, Invasion and Metastasis of Epithelial Cells, Max Delbrück Center of Molecular Medicine, Berlin, Germany

**Keywords:** Clear cell renal cell carcinoma, piRNA, Prognosis, Metastasis, Recurrence, Overall survival

## Abstract

**Background:**

Piwi-interacting RNAs (piRNAs) are small RNAs of 27–30 nucleotides mapping to transposons or clustering in repeat genomic regions. Preliminary studies suggest an important role in cancerogenesis. This study is the first one investigating their prognostic impact in clear cell renal cell cancer (ccRCC) patients.

**Methods:**

Three piRNAs (piR-30924, piR-57125, and piR-38756) selected on the basis of initial piRNA microarray analyses were determined using RT-qPCR in non-metastatic (n = 76) and metastatic (n = 30) ccRCC tissue at the time of nephrectomy in comparison to normal renal tissue (n = 77) and tissue from distant ccRCC metastases (n = 13). Primary clinical end points were recurrence-free and overall survival.

**Results:**

piR-57125 showed lower expression in metastatic than in non-metastatic tumors, whereas the expression of piR-30924 and piR-38756 increased in metastatic tumors. The higher expression of piR-30924 and piR-38756 as well as the lower expression of piR-57125 in metastatic primary tumors were significantly associated with tumor recurrence and overall survival. Multivariate Cox regression analyses revealed both piR-30924 and piR-57125 as independent prognostic predictors. This impact was even more pronounced in non-metastatic patients.

**Conclusions:**

This study demonstrates that the expression levels of these piRNAs in primary non-metastatic and metastatic ccRCC tissue can serve as potential prognostic biomarkers in combination with clinicopathological factors.

**Electronic supplementary material:**

The online version of this article (doi:10.1186/s13046-015-0180-3) contains supplementary material, which is available to authorized users.

## Introduction

Renal cell cancer (RCC) comprises 2.4 % of all adult malignancies worldwide and is one of the ten most frequent cancers with a continuously increasing incidence of 2.5 % expected, accompanied with one of the highest cancer-specific mortality rates [1, 2]. Worldwide, RCC accounted for 338 000 new cases and 144 000 deaths in 2012. Incidence rates of 6 per 100 000 in men and 3 per 100 000 in women and corresponding mortality rates of 2.5 and 1.2 per 100 000, respectively have estimated [1]. It is important from the clinical point of view to distinguish between different histological RCC subtypes [3]. Clear cell RCC (ccRCC) is the most common histological subtype accounting for approximately 80–90 % of all RCCs, followed by papillary RCC at 6–15 % and chromophobe RCC at 2–5 %. The majority of participants at the International Society of Urological Pathology consensus conference 2012 agreed that the main morphotypes of RCC are significant prognosticators [4]. In text books and also guidelines, a significantly worse prognosis is outlined for patients with ccRCC after nephrectomy compared with patients suffering from papillary or chromophobe RCC [3, 5]. However, the study results are not at all consistent [6, 7]. It should be considered that patients with papillary RCC type 1 have a more favorable outcome than those with type 2 [8]. This feature correspond to the observation that two subgroups of papillary RCC can be distinguished, one with a better and another with a worse prognosis in comparison to ccRCC [7]. After a curatively intended nephrectomy, approximately 30 % of RCC patients develop metastases with an average survival time of about 24 months [9]. Thus, there is an urgent need for a better prediction of high risk RCC patients to apply potential personalized therapeutic strategies [10]. However, current prognostic models based on conventional clinicopathological and imaging data have limited accuracy and need further improvements [11]. New molecular markers might be helpful for improving not only diagnosis, but also risk assessment and prediction of the therapeutic response in RCC patients [10–12].

In this respect, small non-coding RNAs like microRNAs (miRNAs) have attracted special attention because of their role as key regulators of gene expression [13]. Another novel class of small RNAs, termed Piwi-interacting RNAs (piRNAs) has essential functions in stem cell division, apoptosis, and epigenetic control of transposons and telomers, but also in translational regulation [14]. These RNAs were first discovered in the testis [15, 16]. In contrast to miRNAs with approximately 20–24 nucleotides, the length of piRNAs is about 26–33 nucleotides. Recently, 32 194 and 32 826 piRNA sequences have been listed in the two most comprehensive piRNA data bases (http://pirnabank.ibab.ac.in/; http://www.regulatoryrna.org/database/piRNA/) [17, 18]. Seventy to eighty percent of all these piRNA sequences have been found on unique genomic loci with the remaining 20*–*30 % on multiple genomic loci. Early studies on different tumor types like gastric, colon, lung, and breast cancer showed that dysregulated piRNAs can be involved, just like miRNAs, in cancerogenesis [19–25]. However, in ccRCCs as the most frequent RCC, no studies have yet been conducted in view of the profile of piRNAs and their potential use as diagnostic and prognostic biomarkers in this cancer.

Therefore, the aims of this study were (a) to characterize the piRNA expression profile in ccRCC using an exploratory microarray technology, (b) to investigate three piRNAs selected on the basis of microarray results as differentially expressed examples and to evaluate the expression profiles in non-metastatic and metastatic primary ccRRCC in comparison to normal renal parenchyma distant from the tumor [26] or tissue from distant ccRCC metastases [27] using quantitative real-time reverse-transcription polymerase chain reaction (RT-qPCR), (c) to estimate the possible associations between expression patterns and clinicopathological data, and (d) to characterize their prognostic potential with regard to tumor recurrence and overall survival.

## Materials and methods

The study was approved by the institutional Ethics Committee (EA1/153/07; EA1/134/12) and was conducted in compliance with the declaration of Helsinki. Reporting in this study follows the REMARK criteria [28].

### Patients and tissue samples

The study included 106 patients undergoing radical nephrectomy for non-metastatic or metastatic primary ccRCC between 2003 and 2010. In this study group, there was radiological evidence of metastases in 30 patients at the time of surgery while 76 patients were free of metastases. This was a retrospective study. The number of patients was based on sample size calculations to reach a sufficient power of this study (α = 5 %, β = 80 %) as explained in Additional file 1: Supporting Information S1. Samples collected in the above mentioned period were then selected at random according to the availability of cryo-preserved tissue. Tumor tissue and normal renal parenchyma distant from the tumor, as recently suggested as sampling approach for nephrectomy samples [26, 29], were immediately sampled after nephrectomy, either snap frozen in liquid nitrogen or immersed in RNA Later solution (Qiagen, Hilden, Germany) and stored at −80 °C as previously described [30, 31]. We defined a necessary distance of >20 mm to the cancer tissue to be absolutely asure of lowest possible alteration of the non-neoplastic tissue through the tumor. That would also take into account the theory of the field effect that cells in the proximity to cancer could exhibit characteristics of the cancer cells [32]. Thus, missing normal tissue samples due to large tumor sizes of some patients omitting normal tissue sampling or other procedure associated reasons as recommended by Srigley et al. [29] resulted in 77 normal and 106 tumor samples. Additionally, 13 bone metastatic ccRCC specimens were sampled. No patients had received systemic therapy prior to tissue collection. The tumors were classified according to the 2002 TNM classification and the Fuhrman grading system by an experienced pathologist (EK) [33, 34].

### RNA extraction

Isolation of total RNA, its quantification and quality characteristics were performed as previously reported [30, 31]. Frozen histologic sections, stained with hematoxylin/eosin, were prepared from the stored tissue samples for the extraction of total RNA. Only samples with at least 80 % tumor cells and without significant areas of necrosis or fibrosis were used. About 30–60 mg of tissue pieces were disrupted in QIAzol Lysis Reagent (Qiagen) with 5 mm stainless steel beads using the TissueLyser System (Qiagen). Homogenates were used for the isolation of total RNA using the miRNeasy Mini Kit (Qiagen) including an optional on-column DNA digestion step according to the producer’s instructions. Total RNA was eluted from the spin column after different washing steps with 30 μl RNase-free water. In this way, the isolated total RNA also included all RNAs <200 nt. RNA samples were spectrophotometrically quantified and characterized by the ratio of the absorbance measured at 260 nm to that at 280 nm (NanoDrop Technologies, Wilmington, DE, USA) and by the RNA integrity number using a Bioanalyzer 2100 (Agilent Technologies, Santa Clara, CA, USA) with a RNA 6000 Nano Lab Chip, as previously described [30]. The median ratio of 260 nm to 280 nm and the median RNA integrity number of all the isolated RNA samples were 2.03 (95 % CI, 2.03*–*2.04) and 7.65 (95 % CI, 7.40*–*7.80), respectively. The median RNA yield from one mg wet weight of tissue was 716 ng (95 % CI, 664–777 ng).

### Microarray analysis and selection criteria of piRNA for further analysis

Three RNA pools containing equal amounts of total RNA from five different non-metastatic ccRCC tissue samples and five different normal tissue samples as defined above were examined as a custom order by ArrayStar Inc., Rockville, MD, USA. For that purpose, one microgram of each pooled sample was 3′-end-fluorescently labelled with Cy3. The labelled samples were hybridized on the ArrayStar HG19 piRNA microarray that was designed for profiling of about 23 000 human piRNAs. The array images and subsequent data were analyzed using software from Agilent Technologies, Santa Clara, CA, USA (Agilent Feature Extraction software, version 10.7.3.1; GeneSpring GX v11.5.1 software package). Quantile normalization of data was applied. Differentially expressed piRNAs were identified using a Volcano plot (Additional file 1: Supporting Information S2 and Figure S1) and the individual data are listed in a separate Excel file (Additional file 2: Supporting Information Excel file). We defined as selection criteria for the further quantitative validation of piRNAs using RT-qPCR a differential expression of fold change >3 with *p*-values <0.005 and a high intensity (raw intensity in the chip analysis >700). Thus, the upregulated piR-38756 and the downregulated piR-57125 were selected for further analysis. piR-30924 was additionally included in this program (Additional file 1: Supporting Information Figure S1) since this piRNA was shown in one of the first clinically oriented piRNA studies in cancer to be a useful tumor marker [20]. In the following, we used the piRNA names of the data base of the National Center for Biotechnology Information, Rockville, USA (http://www.ncbi.nlm.nih.gov/) instead of the long NCBI accession numbers or the accession numbers of the other mentioned data bases [17, 18].

### Quantitative RT-PCR

piR-30924, piR-38756, and the two miRNAs miR-28 and miR-106a as reference genes were determined according to the principle of the TaqMan RT-qPCR assay (Applied Biosystems, Foster City, CA), as previously described [30, 35, 36], while piR-57125 was determined using the miScript PCR system (Qiagen, Hilden, Germany). Customized assays were used for piRNA measurements. All methodical details including the MIQE guideline checklist [37], the PCR product controls, and precision control data were compiled in the Additional file 1: Supporting Information S3 including the Tables S2-S5 and Figure S2. qPCR measurements were performed on the Light-Cycler 480 (Roche, Mannheim, Germany).

### Data analysis and statistics

RT-qPCR data were analyzed using the software qBasePLUS, version 2.6 (Biogazelle, Zwijnaarde, Belgium). Statistical analyses were performed with SPSS 21 (SPSS Inc., Chicago, IL) using bootstrapping calculations, GraphPad Prism 6.05 (GraphPad Software, La Jolla, CA), and MedCalc 14.8.1 (MedCalc Software, Ostend, Belgium). The statistical tests (non-parametric: Mann–Whitney *U-*test, Kruskal-Wallis test, Spearman rank correlation; parametric: ANOVA, Student’s *t*-tests with log-transformed data) are mentioned in the corresponding places. Receiver operator characteristics (ROCs) with areas under the curve (AUCs) and binary logistic regression served to identify the discriminating capacity of piRNAs. The Kaplan-Meier approach and Cox proportional hazard regression analysis were used for disease progression analyses (overall survival and recurrence-free survival). The C-index was calculated as a global measure for validating the predictive reliability of survival models [38]. *P* < 0.05 (two-sided) was considered statistically significant. GraphPad Statmate 2.0 (GraphPad Software) and MedCalc were used for sample size determinations (α = 5 %, β = 80 %) as explained in detail in Additional file 1: Supporting Information S1.

## Results

### Patient characteristics

The clinicopathological characteristics of the study cohorts are compiled in Table 1. Thirteen metastatic ccRCC specimens from bone metastases (12 male, one female; median age: 69 years; range: 40–89) were additionally investigated to compare the expression characteristics of piRNAs.

**Table 1:** Cohort characteristics, *N* = 106

Characteristics	Primary ccRCC, non-metastatic^a^ N = 76	Primary ccRCC, metastatic^a^ *N* = 30	*P* value^b^
Age, median years (range)	65 (37–87)	61 (43–76)	0.153
Sex, n (%)			
Male	58 (76)	21 (70)	0.621
Female	18 (24)	9 (30)	
Pathological stage, n (%)			
pT1	48 (63)	5 (17)	<0.0001
pT2	2 (3)	1 (3)	
pT3	25 (33)	21 (70)	
pT4	1 (1)	3 (10)	
Fuhrman grade, n (%)			
G1	11 (14)	0 (0)	<0.0001
G2	60 (79)	11 (37)	
G3	3 (4)	16 (53)	
G4	2 (3)	3 (10)	
Surgical margins, n (%)			
R0	71 (93)	21 (70)	0.003
R1/2	5 (7)	9 (30)	
Tumor size, median mm (range)	48 (20–180)	85 (35–170)	<0.0001
Patients followed,^c^ n (%)	76 (100)	30 (100)	<0.0001
Metastasis at follow-up	17 (29)		
Death at follow-up	12 (16)	25 (83)	
Follow-up, median month (range)			
Overall	65.7 (1.0*–*121)	9.5 (1.2*–*65.2)	<0.0001
No recurrence	69.9 (5.3*–*121)		
Recurrence	30.4 (1.0*–*116)		

*Abbreviations*: *ccRCC* clear cell renal cell carcinoma, *G* histopathological grading according to Fuhrman, *pT* pathological tumor classification, *R* surgical margin classification

^**a**^Imaging techniques was used to provide evidence of presence/non-presence of metastases before surgery

^**b**^ *p* value from Fisher’s exact test or Chi-square test for trend and Mann–Whitney *U*-test

^**c**^In four cases, only the survival but not the recurrence situation could be assessed

### Differential expression of piRNAs and their relation to clinicopathological data

235 upregulated and 369 downregulated piRNAs of 23 677 piRNAs on the microarray (>2 fold change, *p* < 0.05; details in Additional file 1: Supporting Information S2) were found in malignant in comparison to non-malignant tissue. According to the above mentioned selection criteria for further RT-qPCR analyses of piRNAs and to obtain deeper insight into their differential expression in ccRCC, we selected piR-38756, piR-57125, and piR-30924 measurements in individual tissue samples of the study groups.

**Fig. 1:**
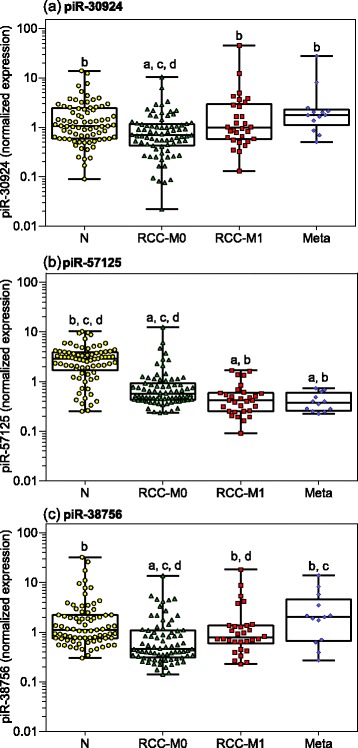
Expression of piRNAs in tissue samples from patients suffering from clear cell renal cell carcinoma. Measurements of (**a**) piR-30924, (**b**) piR-57125, and (**c**) piR-38756 were performed in normal renal parenchyma distant from tumor (N, *n* = 77), in tissue from non-metastatic (RCC-M0, *n* = 76) and metastatic primary tumor (RCC-M1, *n* = 30) of patients with clear cell renal cell carcinoma at the time of nephrectomy as well as in bone metastatic tissue samples (Meta, *n* = 13) from patients suffering from renal cell carcinoma metastases. The miRNAs miR-28 and miR-106a were used as normalizers according to previous results on suitable reference genes for miRNA expression in renal cell carcinomas [30]. Boxes in the box-and whisker plots represent the lower and upper quartiles with medians, whiskers illustrate the whole range of the samples. Significant differences between the study groups were estimated by the one-way ANOVA test with multiple comparisons corrected according to Holm-Sidak. Significances of at least *p* < 0.05 are indicated by the following symbols: a, compared to “N”; b, compared “RCC-M0”; c, compared to “RCC-M1”, and d, compared to “Meta”

In Fig. 1, the RT-qPCR expression data of these three piRNAs in normal tissue, non-metastatic and metastatic primary tumor samples as well as in bone metastatic ccRCC specimens are presented. miR-28 and miR-106a were used as normalizers described in Additional file 1: Supporting Information S3. Our previous studies on reference genes for the expression of miRNAs, and thus also for slightly longer piRNAs, in renal cell carcinomas demonstrated the better accuracy of this combination of two miRNAs in comparison to the conventionally used RNU6B or RNU48 [30]. The expression rates of the three piRNAs were not associated with age or sex (r_S_ = 0.006*–*0.151, *p =* 0.151*–*0.938). All three piRNAs had reduced expression in non-metastatic primary tumors in comparison to normal renal tissue. Additionally, piR-57125 was lower in metastatic primary tumors and in bone metastatic tissue compared to non-metastatic primary tumors, while, on the contrary, piR-30924 and piR-38756 were significantly upregulated in metastatic primary tumors and in bone metastases compared to non-metastatic primary tumors. Depending on tumor stage and grade, significantly higher expression rates were found in tumor samples of pT3 + 4 and Fuhrman G3 + 4 compared to those of pT1 + 2 or G1 + 2 for piR-30924 (geometric means of normalized expression of 1.03 vs. 0.544, *p* = 0.008 and 3.43 vs. 0.604, *p* < 0.0001, respectively) and piR-38756 (0.936 vs. 0.513, *p* = 0.024 and 3.23 vs. 0.562, *p* < 0.0001, respectively), but not for piR-57125 (0.682 vs. 0.709, *p* = 0.365 and 0.678 vs. 0.912, *p* = 0.366, respectively) (Additional file 1: Supporting Information Figure S3). Tumor size was significantly correlated only with piR-30924 (r_S_ = 0.305, *p =* 0.007) but not with piR-57125 (r_S_ = 0.001, *p* = 0.989) and piR-38756 (r_S_ = 0.088, *p =* 0.447). Statistically significant associations were not found between the surgical margin and all the piRNAs (*p =* 0.068*–*0.302).

The expression of piR-30924 correlated with piR-57125 and piR-38756 (r_S_ = 0.320, *p* < 0.0001 and r_S_ = 0.578, *p* < 0.0001), but no correlation existed between the expression of piR-57125 and piR-38756 (r_S_ = 0.103, *p =* 0.151).

### piRNAs as marker for tissue discrimination

In Table 2, the ROC analyses of piRNAs as tissue discriminators are summarized. A better discrimination between non-malignant and malignant tissue samples than between metastatic and non-metastatic samples was achieved; the correct classification rates were about 80 %.

**Table 2:** Receiver-characteristic curve analyses of piRNAs to discriminate between non-malignant vs. malignant tissue and non-metastatic vs. metastatic primary tumor tissue

piRNA	AUC (95 % CI)	*P* value different to AUC = 0.5	Differentiating ability at the Youden index^a^		Overall correct classification (%)
			Sensitivity (95 % CI)	Specificity (95 % CI)	
Non-malignant/malignant tissue					
piR-30924	0.61 (0.53*–*0.70)	0.008	75 (65–83)	47 (35–59)	57.3
piR-57125	0.87 (0.81*–*0.92)	<0.0001	90 (82–95)	79 (69–88)	83.6
piR-38756	0.71 (0.64*–*0.78)	<0.0001	43 (33–52)	95 (87–99)	58.5
All piRNAs combined^b^	0.91 (0.86*–*0.95)	<0.0001	91 (83–95)	86 (76–93)	85.8
Non-metastatic/metastatic					
piR-30924	0.63 (0.54*–*0.73)	0.028	30 (15–49)	93 (85–98)	71.7
piR-57125	0.68 (0.58*–*0.77)	0.004	40 (23–59)	92 (84–97)	71.7
piR-38756	0.64 (0.54*–*0.73)	0.013	77 (58–90)	61 (49–72)	71.7
All piRNAs combined^b^	0.76 (0.67*–*0.84)	<0.0001	73 (54–88)	74 (62–83)	75.5
piR-30924 + piR-57125^c^	0.76 (0.67*–*0.84)	<0.0001	73 (54–88)	74 (62–83)	75.5

*Abbreviations*: *AUC* area under the receiver-operating curve, *CI* confidence interval

^**a**^The Youden index as a measure of overall diagnostic effectiveness is calculated by (sensitivity + specificity) - 1. When equal weight is given to sensitivity and specificity of a test, the cutoff at the maximum value of this index, which graphically corresponds to the maximum vertical distance between the ROC curve and the diagonal line, is referred to as optimal criterion

^**b**^Calculated by full binary logistic regression

^**c**^Calculated by stepwise binary logistic regression with all three miRNAs (backward likelihood elimination; entry *p* = 0.05, removal *p* = 0.10). In case of the differentiation between non-malignant and malignant tissue, the backward elimination did not result in a reduced model

### piRNAs as prognostic markers

The RT-qPCR expression data of the piRNAs as shown in Fig. 1 and their association to tumor stage and grade can indeed be considered as an indication that piRNAs could be used as prognostic markers in ccRCC patients. Therefore, we assessed their predictive capacity regarding recurrence and overall survival. The time from the date of surgery to the last follow-up or to tumor recurrence and the determination of death, respectively, were used to calculate the clinical endpoints times of recurrence or overall survival.

**Fig. 2:**
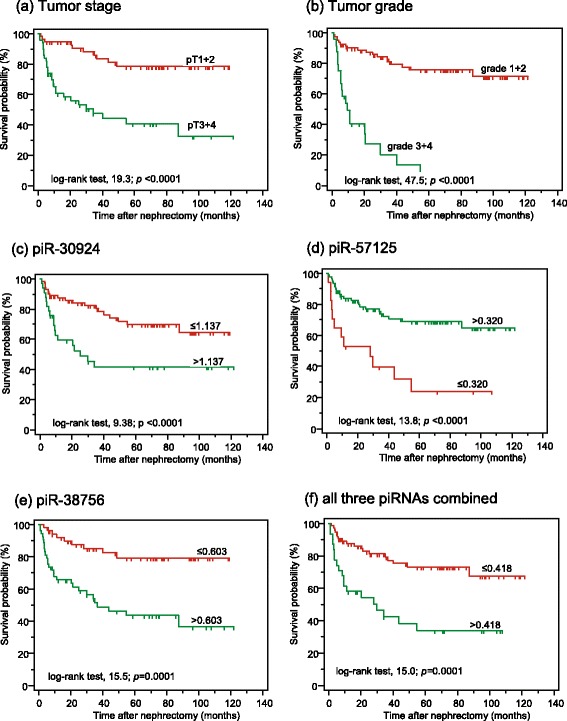
Kaplan-Meier analyses of overall survivals after surgical removal of non-metastatic and metastatic clear cell renal cell carcinoma. piRNAs were dichotomized using their values (given in normalized units) obtained in the ROC analysis at the point of maximal accuracy to discriminate between dead and alive. The curves from 76 non-metastatic and 30 metastatic patients listed in Table 1 are presented according to (**a**) pathological stage, (**b**) grading, and expression of (**c**) piR-30924, (**d**) piR-57125, (**e**) piR-38756, and (**f**) combination of all three piRNAs. The log-rank test was used to confirm significant differences between the survival probabilities

**Fig. 3:**
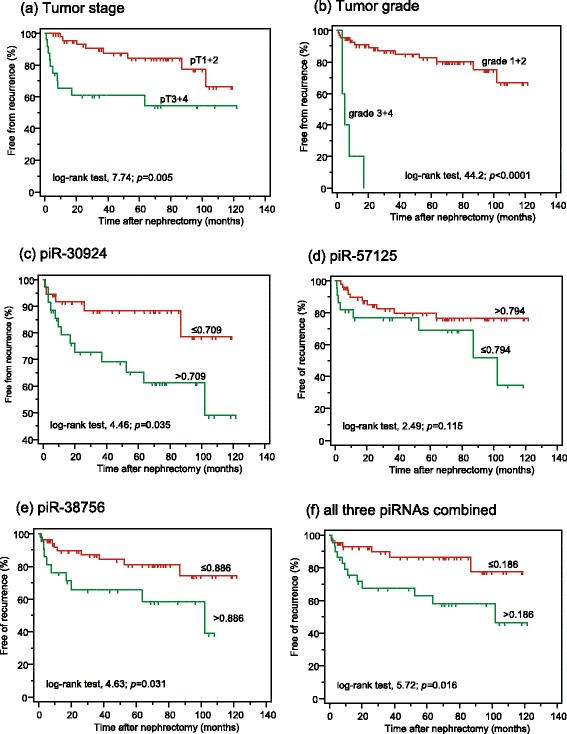
Kaplan-Meier analyses of recurrence-free survivals after surgical removal of non-metastatic clear cell renal cell carcinoma. piRNAs were dichotomized using their values (given in normalized units) obtained in the ROC analysis at the point of maximal accuracy to discriminate between recurrence and recurrence free. The curves include data from 72 of the 76 non-metastatic patients listed in Table 1 with available follow-up data and are presented according to (**a**) pathological stage, (**b**) Fuhrman grading, and expression of (**c**) piR-30924, (**d**) piR-57125, (**e**) piR-38756, and (**f**) combination of all three piRNAs. The log-rank test was used to confirm significant differences between the recurrence-free intervals

To provide an initial overview, Kaplan-Meier curves were calculated to assess the association of piRNA expression data, which were dichotomized using the cutoffs obtained in the ROC analysis at the point of maximal accuracy (Youden index), and clinical outcomes (Figs. 2 and 3). The cutoff values are displayed at the respective curves. The overall survival and the recurrence-free interval significantly decreased with increasing pT stage and histological grade, thus demonstrating the representativeness of the study cohorts. The higher expression of piR-30924 and piR-38756 as well as the lower expression of piR-57125 in metastatic tumors and bone metastases compared to the non-metastatic primary tumors was significantly associated with the two clinical endpoints. Subsequently, the prognostic performance of the individual piRNAs was assessed together with the relevant clinicopathological variables both in univariate and fully and stepwise reduced multivariate Cox regression analyses (Table 3). In univariate analyses, the hazard ratios of piRNAs reflected the results of the Kaplan-Meier curves. For the combined tumor group, including metastatic and non-metastatic patients, the criterion “metastasis” was the decisive factor for overall survival (Table 3). However, the backward elimination approach of the multivariate Cox regression with all clinicopathological factors and piRNAs showed that, in addition to the criterion “metastasis”, both piR-30924 and piR-57125 remained in the model as significant variables. On the other hand, the prognostic significance of piR-38756 was especially indicative in the non-metastatic patients as this piRNA alone remained in the model as essential predictive variable together with the variable “Fuhrman grade”.

**Table 3:** Cox proportional hazard regression analyses of clinicopathological factors and piRNAs for predicting overall and recurrence-free survival in ccRCC patients after nephrectomy and C-indexes of the models

	Overall survival analysis^a^				Recurrence-free survival analysis^a^	
	All ccRCC patients (*N* = 106)		Non-metastatic ccRCC (*N* = 76)		Non-metastatic ccRCC (*N* = 72)	
Variable^b^	HR (95 % CI)	*P* value	HR (95 % CI)	*P* value	HR (95 % CI)	*P* value
Univariate analysis						
Age (continuous)	0.98 (0.96*–*1.01)	0.300	0.99 (0.94*–*1.04)	0.771	1.01 (0.97*–*1.05)	0.709
Gender	0.97 (0.46*–*2.04)	0.929	2.95 (0.39*–*22.8)	0.297	5.35 (0.72*–*39.8)	0.103
pT stage (pT1-2/pT3-4)	4.44 (2.14*–*9.93)	<0.0001	3.57 (1.13*–*11.3)	0.031	3.47 (1.37*–*8.78)	0.009
Grade (G1-2/G3-4)	7.75 (3.91*–*15.4)	<0.0001	17.3 (3.67*–*81.3)	0.003	4.73 (2.27*–*9.84)	<0.0001
Margin (R0/R1-2)	2.51 (1.44*–*4.38)	0.001	3.15 (0.82*–*12.2)	0.098	6.23 (2.66*–*14.6)	<0.0001
Tumor size (continuous)	1.01 (1.01*–*1.02)	<0.0001	1.01 (1.01*–*1.02)	0.020	1.01 (1.01*–*1.02)	<0.0001
Metastasis	11.6 (5.60*–*24.2)	<0.0001	-	-	-	-
piR-30924	2.90 (1.36*–*4.94)	0.004	3.11 (1.01*–*3.50)	0.049	2.89 (1.04*–*8.08)	0.044
piR-57125	0.30 (0.15*–*0.59)	0.0006	0.0	0.964	2.08 (0.82*–*5.25)	0.123
piR-38756	3.96 (1.87*–*8.37)	0.0003	5.40 (1.47*–*19.9)	0.012	2.66 (1.06*–*6.67)	0.038
Multivariate analysis^c^						
T stage	0.72 (0.24*–*2.15)	0.718	1.42 (0.30*–*6.81)	0.658	1.57 (0.38*–*6.52)	0.536
Grade	1.17 (0.43*–*3.18)	0.760	8.32 (0.52*–*33.4)	0.135	7.75 (1.11*–*54.4)	0.039
Margin	1.57 (0.67*–*3.69)	0.298	1.08 (0.19*–*6.14)	0.935	2.50 (0.77*–*8.15)	0.127
Tumor size	1.00 (0.99*–*1.02)	0.603	0.99 (0.97*–*1.02)	0.457	1.00 (0.99*–*1.02)	0.743
Metastasis	8.41 (3.10*–*22.8)	<0.0001	-	-		
piR-30924	2.04 (1.02*–*4.51)	0.046	1.10 (0.24*–*5.03)	0.899	1.50 (0.41*–*5.43)	0.540
piR-57125	0.50 (0.21*–*1.18)	0.112	-	-		
piR-38756	1.93 (0.72*–*5.16)	0.190	5.42 (0.99*–*29.8)	0.052	3.15 (1.96*–*9.32)	0.038
C-Index with all variables^d^	0.854		0.759		0.803	
C-Index without piRNAs	0.835		0.589		0.753	
Multivariate analysis, backward elimination^e^						
Metastasis	11.0 (4.82*–*25.3)	<0.0001	-	-	-	-
Grade	-	-	8.00 (1.20*–*53.4)	0.032	27.8 (6.60*–*113)	<0.0001
piR-30924	2.27 (1.13*–*4.58)	0.022	-	-	-	-
piR-57125	0.44 (0.21*–*0.93)	0.031	-	-	-	-
piR-38756	-	-	5.67 (1.14*–*28.1)	0.034	3.22 (1.16*–*8.94)	0.025
C-Index of the model	0.840		0.749		0.741	

*Abbreviations*: *ccRCC* clear cell renal cell carcinoma, *CI* confidence interval, *C-Index* concordance index, *G* histopathological grading according to Fuhrman, *HR* hazard ratio, *pT* pathological tumor classification, *R* surgical margin classification

^**a**^This study group included the two cohorts of non-metastatic (*n* = 76) and metastatic (*n* = 30) patients listed in Table 1; for the analysis of recurrence-free survival, data from four patients could not be obtained

^**b**^Calculations were performed with categorization criteria as indicated in brackets and *p* values were obtained by bootstrapping (2000 resamples). piRNAs were dichotomized using their values obtained in ROC analyses at the point of maximal accuracy to discriminate between dead and alive as well as between recurrence and recurrence-free situation. The thresholds corresponded to those used in the Kaplan-Meier analyses in Figs. 2 and 3

^**c**^The multivariate analysis included all variables of the univariate analyses with *p* values <0.10

^**d**^C-indexes [38] were calculated using models either with all variables or only with clinicopathological data without piRNAs

^**e**^The multivariate analysis with the backward elimination approach was made with *p* = 0.05 for entry and *p =* 0.10 for removal. The 95 % CI of the hazard ratios and the *p* values of the final model were obtained after bootstrapping

Internal validation of the results was performed by bootstrapping and the calculation of the C-index [38]. The C-indexes of the survival models showed both the global prognostic ability of the three piRNAs and their additional benefit if they were included into survival models based only on clinicopathological variables (Table 3).

## Discussion

Within the current study, we performed a genome-wide expression analysis of piRNA by microarray to screen for piRNAs differentially expressed in ccRCC and to validate their potential clinical significance as biomarkers. RT-qPCR analyses on three selected piRNAs showed for the first time differentially expressed piRNAs in ccRCC as well as a strong relationship between piRNA expression data and the clinical endpoints of recurrence-free and overall survival. This study design as a hypothesis-generating approach through discovery-driven global research was recently suggested as one of the essential steps for translational research in medicine to comply with the realistic conditions in clinical settings [39].

We found that 1.56 % (n = 369) of the total 23 677 piRNAs examined were downregulated by least two-fold in ccRCC while only 0.99 % (n = 235) were accordingly upregulated. This significantly higher percentage (*p* < 0.0001) of downregulated piRNAs corresponded to a higher number of decreased miRNAs as another type of small RNAs in ccRCC samples [35]. This result of differentially expressed piRNAs prompted us to analyze three selected piRNAs (piR-30924, piR-57125, and piR-38756) in more detail. Although piR-38756 was overexpressed in microarray analysis, its downregulation was demonstrated by RT-qPCR and verified by specific electrophoretic PCR product controls (Additional file 1: Supporting Information Figure S2). This fact underscores again that specific RT-qPCR measurements remain indispensable for the validation of microarray analyses [40, 41]. Other studies found approximately 6*–*10 % discordant microarray results compared to RT-PCR measurements [40]. Moreover, piR-30924, which we selected for comparative purposes as a strongly upregulated piRNA in other cancers [20] was expressed at a lower level in ccRCC than in normal tissue. This discordant expression behavior between different tumors conforms to a similar phenomenon observed in case of miRNAs, probably as a consequence of their different effects or resulting from specific signaling pathways in various tumors [42].

Although all three selected piRNAs were downregulated in non-metastatic ccRCCs in comparison to normal tissue, they were characterized by different expression patterns in the metastatic primary tumor and metastatic tissue from bone metastases (Fig. 1). The uniformly reduced expression of piR-57125 in the sequence from the non-malignant tissue over the primary non-metastatic and primary metastatic tumor to the distant metastases contrasted with the differently regulated expression of piR-30924 and piR-38756 (Fig. 1). The expression of these two piRNAs in samples from metastatic primary ccRCC and distant ccRCC metastases was higher than in non-metastatic ccRCC samples and reached again the level in normal tissue distant from tumor. These differences of the piRNAs were partly reflected by different expression levels, depending on tumor stage and grade (Additional file 1: Supporting Information Figure S3). Similarly characteristic behavior was also observed for miRNAs in ccRCC [43].

In addition to these particularities, the evidence of the investigated piRNAs as independent factors in multivariate Cox regression analyses together with conventional clinicopathological variables like tumor stage and/or tumor grade demonstrates the potential of piRNAs as orthogonal biomarkers [44]. This type of biomarker is characterized by its uncorrelated differential expression to established disease variables. In this case, piRNAs provide an additional degree of information. Thus, the specific feature of orthogonal markers offers the advantage of discovering associations with new disease-related pathways and downstream conditions that had not been considered previously [44]. For predicting overall survival both in non-metastatic and metastatic ccRCC patients after nephrectomy, all three piRNAs were highly significant indicators in univariate analyses (Table 3). More importantly, both piR-30924 and piR-57125 remained independent factors, together with the decisive variable metastasis, in the Cox regression backward model for these patients (Table 3). piR-38756 alone was an independent factor together with tumor grade for predicting recurrence and survival in non-metastatic patients (Table 3). Thus, these data show that piRNAs might be considered as potential new molecular markers that are capable of improving risk stratification and prediction of the therapeutic response in RCC patients. Recent prognostic models, generally based only on clinicopathological and imaging data, are of limited accuracy [11]. This view and the potential improvement of these models using piRNAs as adjunct biomarkers are also supported in our study by the higher C-indexes of the Cox-regression models if piRNAs were used in combination with standard clinicopathological factors in comparison to models with only clinicopathological variables (Table 3).

Expression studies of piRNAs with a focus on their diagnostic and prognostic validity and their functional role have been examined in only a few other cancers [19–21, 23, 25]. As already mentioned, piR-30924 was found to be upregulated in gastric cancer [20]. Transfection of an inhibitor of this piRNA into gastric cancer cells reduced cell proliferation by arresting cells in the G_2_/M phase. An oncogenic role of this piRNA was assumed. In bladder cancer, 106 up- and 91 down-regulated piRNAs were described when similar differential expression criterion of a >2-fold change was applied, as in our study [25]. The authors used the same piRNA microarray and found piR-60152 as the most profoundly downregulated piRNA. Over-expression of this piRNA in bladder cancer cell lines reduced cell proliferation and promoted apoptosis. TNFSF4 was identified as a target gene of this piRNA. piRNA-mediated effects were also characterized for other genes [23, 45]. The identification of piRNA targets is one of the key challenges in our understanding of the function of this class of small non-coding RNAs. Such findings could lead to novel therapeutic implications of piRNAs. In the case of the three studied piRNAs, piR-30924 is located on chromosome 2 and 5 and piR-57125 on chromosome 5 whereas the sequence of piR-38756 has been found at approximately 300 multiple loci. However, the recently reported comprehensive molecular characterization of ccRCC did not mention any relationships between typical RCC hot spots and piRNAs [46]. In addition, sophisticated search machines for target predictions of human piRNAs like for miRNAs are planned but are currently not available [18, 47].

Nevertheless, with regard to the currently missing functional data, it cannot be distinguished as to whether all these different changes are part of the cause of cancerogenesis and its progression or rather the consequence of other processes taking place in cancer development. Recently, the first manually curated piRNA data base (http://www.regulatoryrna.org/database/piRNA/) was established to provide information from more than 130 data sets with regard to the functions of piRNAs in the epigenetic and post-transcriptional regulation of transposons and genes [18]. However, the hitherto scarce insight into the functional role of piRNAs was shown by the quality score <10 of most of the piRNAs in the data base “GeneCards”, including those measured in this study [48]. In this data base that covers the most comprehensive list of approximately 80 000 non-redundant non-coding RNAs including the piRNA class, this low score value characterizes missing functional data and reliable expression profiling studies. All these facts highlight that our understanding of piRNAs is still very limited. Thus, although research on piRNAs is still very much in its infancy and their functional role remains widely unknown [49], these few first results suggest that piRNAs could gain a comparable importance for cancer research in the future as miRNAs achieved some years before [50].

Some limitations of this study, like its retrospective design, the limited number of cases, and the small number of analyzed piRNAs as well as the single-center character of our analysis need to be addressed. Another critical point was the missing normal tissue samples from all nephrectomy samples. We explained it in the section “Materials and methods”. Thus, statistical tests for matched pairs samples could not be performed. In comparison to matched paired samples that consider the intra-individual variability, the variability in groups is generally higher and results in lower statistical power when differences of group data are statistically tested. On the other hand, statistically significant differences detected between groups, as in our study, are consequently a more stringent proof of excluding a possible type II error. Further arguments for the validity of our study are that all measurements were performed in a blinded manner. In addition, at least 10 % more patients were analyzed than the calculated sample size and an internal validation by bootstrapping was performed to avoid type I and II errors. Despite consideration of all these reasonable points, our study results need to be validated by larger multicenter, prospective studies. In such a study including sufficient cases for separate training and test sets, more highly differentially expressed piRNAs as potential predictive and prognostic biomarkers should be examined.

In conclusion, the analysis of piRNAs in ccRCC samples based on genome-wide microarray and RT-qPCR measurements demonstrated their strong association with tumor progression, suggesting in their validity as diagnostic and especially prognostic biomarkers. In combination with standard clinicopathological data, piR-30924, piR-57125, and piR-38756 have the potential to improve prognostic information for ccRCC patients. The results of this study make further prospective studies worthwhile as a high number of differentially expressed piRNAs were found.

## Additional files

Additional file 1:
**Supporting Information S1.** Sample size calculations and study design; **Supporting Information S2.** Microarray data, selected piRNAs, Volcano Plot with **Supplemental Table S1.** (Names, accession nos. of the selected piRNAs in RT-qPCR) and **Supplemental Figure S1.** (Volcano plot); **Supporting Information S3.** RT-qPCR methodology with **Supplemental Table S2.** (MIQE checklist according to Bustin et al.), **Supplemental Table S3.** (TaqMan MicroRNA assays for the two reference genes), Quantification of piRNAs (Determination of piR-30924, piR-57125, and piR-38756), Performance data of the RT-qPCR analyses with **Supplemental Figure S2.** (Specificity of the RT-PCR products of piRNA analyses), **Supplemental Table S4.** (Characteristics of the PCR standard curves), and **Supplemental Table S5.** (Intra- and inter-run precision data of qPCR measurements); **Supporting Information S4.** with **Supplemental Figure S3.** (piRNAs in association to tumour stage and grade). Additional file 2:
**Supporting Information Excel file with differentially expressed piRNAs identified using the ArrayStar HG19 piRNA microarray.**

## Abbreviations

AUC: Area under the receiver operating curve
ccRCC: Clear cell renal cell carcinoma
CI: Confidence interval
piRNA: Piwi-interacting RNA
RT-qPCR: Quantitative real time reverse transcription polymerase chain reaction
