# Supplementary material for: Piwi-interacting RNAs as novel prognostic markers in clear cell renal cell carcinomas
Source: J Exp Clin Cancer Res. 2015 Jun 14;34(1):61. doi: 10.1186/s13046-015-0180-3 (PMC4467205; doi:10.1186/s13046-015-0180-3)
Supplement: Additional file 1: — Supporting Information S1. Sample size calculations and study design; Supporting Information S2. Microarray data, selected piRNAs, Volcano Plot with Supplemental Table S1. (Names, accession nos. of the selected piRNAs in RT-qPCR) and Supplemental Figure S1. (Volcano plot); Supporting Information S3. RT-qPCR methodology with Supplemental Table S2. (MIQE checklist according to Bustin et al.), Supplemental Table S3. (TaqMan MicroRNA assays for the two reference genes), Quantification of piRNAs (Determination of piR-30924, piR-57125, and piR-38756), Performance data of the RT-qPCR analyses with Supplemental Figure S2. (Specificity of the RT-PCR products of piRNA analyses), Supplemental Table S4. (Characteristics of the PCR standard curves), and Supplemental Table S5. (Intra- and inter-run precision data of qPCR measurements); Supporting Information S4. with Supplemental Figure S3. (piRNAs in association to tumour stage and grade). [file 13046_2015_180_MOESM1_ESM.docx]

**Supporting Information to**

**Piwi-interacting RNAs as novel prognostic markers in clear cell renal cell carcinomas**

Jonas Busch^1#^, Bernhard Ralla^1#^, Monika Jung^1^, Zofia Wotschofsky^1,2^, Elena Trujillo-Arribas^3^, Philipp Schwabe^4^, Ergin Kilic^5^, Annika Fendler^1,2,6^, Klaus Jung^1,2^*

^1^Department of Urology, University Hospital Charité, Berlin, Germany; ^2^Berlin Institute for Urologic Research, Berlin, Germany; ^3^Laboratorio clinico, Hospital Universitario Virgen Del Rocio, Sevilla, Spain; ^4^Center for Musculoskeletal Surgery, University Hospital Charité, Berlin, Germany; ^5^Institute of Pathology, University Hospital Charité, Berlin, Germany and ^6^Department of Signal Transduction, Invasion and Metastasis of Epithelial Cells, Max Delbrück Center of Molecular Medicine, Berlin, Germany

**Content of tHe supporting Information**

[Supporting Information S1: Sample size calculations and study design 2](#_Toc409480311)

[Supporting Information S2: Microarray data, selected piRNAs, Volcano Plot 2](#_Toc409480312)

[Supplemental Table S1. Names, accession nos. of the selected piRNAs in RT-qPCR 3](#_Toc409480313)

[Supplemental Figure S1. Volcano plot 4](#_Toc409480314)

[Supporting Information S3: RT-qPCR Methodology 5](#_Toc409480315)

[*General comments* 5](#_Toc409480316)

[Supplemental Table S2. MIQE checklist according to Bustin et al. 5](#_Toc409480317)

[*Quantification of miR-28 and miR-106a as reference genes* 7](#_Toc409480318)

[Supplemental Table S3. TaqMan MicroRNA assays for the two reference genes 8](#_Toc409480319)

[*Quantification of piRNAs* 8](#_Toc409480320)

[Determination of piR-30924. 9](#_Toc409480321)

[Determination of piR-57125 9](#_Toc409480322)

[Determination of piR-38756 9](#_Toc409480323)

[*Performance data of the RT-qPCR analyses* 10](#_Toc409480324)

[Supplemental Figure S2. Specificity of the RT-PCR products of piRNA analyses 10](#_Toc409480325)

[Supplemental Table S4. Characteristics of the PCR standard curves 12](#_Toc409480326)

[Supplemental Table S5. Intra- and inter-run precision data of qPCR measurements 12](#_Toc409480327)

[SUPPORTING INFORMATION S4: Additional figures 13](#_Toc409480328)

[Supplemental Fig. S3. piRNAs in association to tumour stage and grade 13](#_Toc409480329)

[References in the Supporting Information 13](#_Toc409480330)

**Separate Excel file: Supporting Information Excel file**

**Supporting Information S1: Sample size and power calculations, study design**

Clinical endpoints with regard to the use of piRNAs as diagnostic and prognostic markers were defined and the necessary sample size was calculated.

In case of piRNAs as **diagnostic markers**, their usefulness to differentiate between malignant and non-malignant tissue was of primary interest. As shown in our other nucleic acid-based expression studies regarding renal cell carcinomas, a difference of 0.8 to one standard deviation could be considered as relevant for the discrimination between the mean expression levels of both tissue types [1]. Thus, to avoid both type I and type II errors the conventional thresholds of α=5% (significance level) and β=20% (1-power; power of 80%) were selected for sample size calculation. Under these conditions, a sample size of 17 to 26 in each group would be necessary to detect piRNA changes. As additional approach, the discrimination capacity corresponding to an AUC value of 0.7 to 0.75 was selected. Under these conditions and again at α=5% and β=20% for type I and II errors, the necessary sample size would be 19 to 30 for each tissue type. To test the ability of tissue piRNAs as tools to discriminate between non-metastatic and metastatic state at the time of nephrectomy, we also considered our data obtained in our previous miRNA studies between metastatic and non-metastatic RCC patients for the calculation of an acceptable sample size [1]. Taking into account relative standard variations between 50 to 100% in metastatic and non-metastatic samples and a difference of about 0.8 standard deviation between the mean expressions of both cohorts as significant, expression differences could be expected by studying of about 25 samples in each patient group at a ratio of 1:1 or a total of 68 samples at a ratio of 1:4 (16 with and 64 without metastases).

In case of piRNAs as **prognostic markers**, two clinical endpoints were of interest, the recurrence and the survival. The necessary sample size for studying the usefulness of piRNAs as predictors of recurrence was calculated considering a proportional difference of progression-free survival of 0.40 in the Kaplan-Meier curves between patients with localized or locally advanced compared to those with metastatic disease [2]. In this respect and considering α=5% and β=20%, about 26 subjects in each group would have to be investigated to avoid type I and type II errors. However, an unequal sample size at a ratio of 1:4 between the groups of tumour recurrence or no recurrence would be possible without any loss of statistical power, if the total sample size was increased to 72 (18 with recurrence and 54 subjects without, respectively). Similar calculations would apply for the survival. The above mentioned sample size calculation for the differentiation between tissue samples from non-metastatic and metastatic RCC patients as "indication of progression" shows a comparable necessary sample size.

Thus, in order to meet all these requirements we decided to include in our study about 70 non-metastatic and 30 metastatic RCC patients at the time of nephrectomy for studying diagnostic (malignant vs. non-malignant issue; non-metastatic vs. metastatic tissue) and prognostic capacity (recurrence/mortality risk).

Supporting Information S2: Microarray data, description of the selected piRNAs (Table S1), and Volcano Plot (Fig. S2)

As described in Materials and Methods, microarray analyses were performed as custom order by ArrayStar Inc., Rockville, MD, USA. Differentially expressed piRNAs (fold-change >1.5 between ccRCC vs normal adjacent tissue, t-test, p-value <0.1) are listed in a separate Supporting Information Excel file that also provides annotations to each probe including description, organism, piRNA accession number, piRNA length and sequence. ArrayStar used as probe name the piRNA accession number (DQ followed by six figures) of the NCBI data base with the added letter "P". Different accession nos. and names of piRNAs are used in literature depending on the data base (e.g., NCBI, National Center for Biotechnology Information, Rockville, USA; <http://www.ncbi.nlm.nih.gov/>). As mentioned in the main text, we used for better legibility and clarification the selected short forms of the NCBI data base instead of the long NCBI accession numbers or the piRNA accession/ID nos. of other data bases. For the selected three piRNAs piR-30924, piR-57125, and piR-38756 in the RT-qPCR measurements, their names/accession numbers in the various data bases are listed in the following Supplemental Tables S1A+B.

**Supplemental Table S1.** Names and accession numbers of the selected piRNAs in RT-qPCR analyses in relation to the piRNA accession/ID nos. in other data bases

A) With regard to the NCBI Genbank

| **Assay name used in**  **manuscript** | **Accession No.**  **(NCBI Genbank)** | **piRNA Name (NCBI Genbank)** | **Target Sequence** |
| --- | --- | --- | --- |
| piR-30924 | DQ570812.1 | piR-30924 | agagaggggcccgtgccttggaaagcgtc |
| piR-57125 | DQ590013.1 | piR-57125 | tggtcgtggttgtagtccgtgcgagaa |
| piR-38756 | DQ600690.1 | piR-38756 | tactttgggaggctgaggcgggtggat |

B) The three examined piRNAs termed on the basis of the NCBI accession number and NCBI piRNA name with respect to the IDs in some other piRNA data bases

| **Data base and reference** | **URL** | **piRNA ID in the data base** |
| --- | --- | --- |
| **NCBI Genbank** | <http://www.ncbi.nlm.nih.gov/> | 1) DQ570812  2) DQ590013  3) DQ600690 |
| **IBAB piRNA bank [3]** | http://pirnabank.ibab.ac.in/ | 1) hsa_piR_00065  2) hsa_piR_014620  3) hsa_piR_022437 |
| **piRBase [4]** | http://[www.regulatoryrna.org/database/piRNA/](http://www.regulatoryrna.org/database/piRNA/) | 1) piR-hsa-1077  2) piR-hsa-20266  3) piR-hsa-30978 |
| **piRNAQuest [5]** | http://bicresources.jcbose.ac.in/zhumur/pirnaquest | 1) hsa_piRNA_31147  2) hsa_piRNA_4946  3) hsa_piRNA_23315 |
| **GeneCards [6]**  **V3.12** | http://www.genecards.org | 1) PIR31923  PIR57642  2) PIR51124  3) PIR61801 |
| **proTRAC [7]** | http://www.uni-mainz.de/FB/Biologie/Anthropologie/492_DEU_HTML.php |  |
| **Assay name**  **in manuscript** |  | 1) piR-30924  2) piR-57125  3) piR-38756 |

The differential expression of piRNAs (fold-change >1.5 between ccRCC vs normal adjacent tissue, t-test, p-value <0.1) is shown in the following Supplemental Fig. S1.

**Supplemental Figure S1. Volcano plot** **of the expression of piRNAs in non-metastatic primary ccRCC tissue. Up- and down-regulated piRNAs are shown in relation to normal adjacent tissue. Vertical and horizontal dashed lines indicate the thresholds of the 1.5 fold changes and the *p*-values of 0.10 in the t-test. The positions of the three detailed examined piRNAs are indicated.**

Using the criteria >1.5 fold change with p values <0.1 (as shown in the Fig. S1) and >2 or >3 fold change with p values <0.05, 903, 235, and 89 piRNAs were found to be upregulated and 1055, 369, and 145, respectively were downregulated. The criterion 3 fold change with p value of 0.005 resulted in 13 upregulated and 62 downregulated piRNAs.

**Supporting Information S3: RT-qPCR Methodology**

***General comments***

**RT-qPCR measurements were performed according to the recommendations of the MIQE guidelines [8]. No template controls (NTC) and no reverse transcription controls (NRTC or no enzyme controls=NEC) were always performed and showed negative results. The corresponding comments are listed in the following checklist (Supplemental Table S2) and apply for all assays.**

**Supplemental Table S2.** MIQE checklist according to Bustin et al. **[8]**

| **ITEM TO CHECK** | **IMPORTANCE** | **CHECKLIST** | **WHERE; COMMENT** |
| --- | --- | --- | --- |
| **Experiemental design** |  |  |  |
| Definition of experimental and control groups | **E** | Yes | Main text: Materials and Methods: Patients and tissue samples, Table 1 |
| Number within each group | **E** | Yes | Main text: Materials and Methods: Patients and tissue samples, Table 1 |
| Assay carried out by core lab or investigator's lab? | D | Yes | Investigator's lab |
| Acknowledgement of authors' contributions | D | No |  |
| **SAMPLE** |  |  |  |
| Description | **E** | Yes | Main text: Materials and Methods: Patients and tissue samples; RNA extraction |
| Volume/mass of sample processed | D | Yes | Main text: Materials and Methods: RNA extraction |
| Microdissection or macrodissection | **E** | Yes | Main text: Materials and Methods: RNA extraction |
| Processing procedure | **E** | Yes | Main text: Materials and Methods: Patients and tissue samples; RNA extraction |
| If frozen - how and how quickly? | **E** | Yes | Main text: Materials and Methods: Patients and tissue samples |
| If fixed - with what, how quickly? | **E** | Not applicable |  |
| Sample storage conditions and duration (esp. for FFPE samples) | **E** | Yes | Main text: Materials and Methods: Patients and tissue samples |
| **Nucleic acid extraction** |  |  |  |
| Procedure and/or instrumentation | **E** | Yes | Main text: Materials and Methods: RNA extraction |
| Name of kit and details of any modifications | **E** | Yes | Main text: Materials and Methods: RNA extraction |
| Source of additional reagents used | D | Not applicable |  |
| Details of DNase or RNAse treatment | **E** | Yes | Main text: Materials and Methods: RNA extraction, on-column DNAse digestion: |
| Contamination assessment (DNA or RNA) | **E** | Yes | Supporting Information S3: RT-PCR methodology: genomic DNA contamination was excluded by control experiments without reverse transcription for all targets; for miRNAs: not affected by genomic DNA contamination [ref. [9]] |
| Nucleic acid quantification | **E** | Yes | Main text: Materials and Methods: RNA extraction |
| Instrument and method | **E** | Yes | Main text: Materials and Methods: RNA extraction |
| Purity (A260/A280) | D | Yes | Main text: Materials and Methods: RNA extraction |
| Yield | D | Yes | Main text: Materials and Methods: RNA extraction |
| RNA integrity method/instrument | **E** | Yes | Main text: Materials and Methods: RNA extraction (RIN; Agilent) |
| RIN/RQI or Cq of 3' and 5' transcripts | **E** | Yes | Main text: Materials and Methods: RNA extraction (RIN; Agilent) |
| Electrophoresis traces | D | No |  |
| Inhibition testing (Cq dilutions, spike or other) | **E** | Yes | Supporting Information S3: RT-qPCR methodology, , Cq dilution, see standard standard curve characteristics in Table S4 |
| **REVERSE transcription** |  |  |  |
| Complete reaction conditions | **E** | Yes | Main text: Material and Methods:Quantitative RT-qPCR; Supporting Information S3: RT-qPCR methodology |
| Amount of RNA and reaction volume | **E** | Yes | Supporting Information S3: RT-qPCR methodology |
| Priming oligonucleotide (if using GSP) and concentration | **E** | Yes | Supporting Information S3: RT-qPCR methodology |
| Reverse transcriptase and concentration | **E** | Yes | Supporting Information S3: RT-qPCR methodology |
| Temperature and time | **E** | Yes | Supporting Information S3: RT-qPCR methodology |
| Manufacturer of reagents and catalogue numbers | D | Yes | Supporting Information S3: RT-qPCR methodology |
| Cqs with and without RT | D | Yes | Supporting Information S3: RT-qPCR methodology: neg. results; see also comment on DNase treatment |
| Storage conditions of cDNA | D | Yes | Supporting Information S3: RT-qPCR methodology; storage at -20°C |
| **qPCR target information** |  |  |  |
| Gene symbols | **E** | Yes | Main text: Materials and Methods: Microarray analysis; Supporting Information S2 |
| If multiplex, efficiency and LOD of each assay. | **E** | Not applicable |  |
| Sequence accession number | **E** | Yes | Supporting Information S2: Table S1, S3 |
| Location of amplicon | D | Yes |  |
| Amplicon length | **E** | Yes | Supporting Information S2: Table S1; Supporting Information: RT-qPCR methodology, Table S3 |
| *In silico* specificity screen (BLAST, etc) | **E** | Yes | Supporting Information S3: RT-qPCR methodology: controlled by the manufacturers (Qiagen, TIBMolBiol, Life technology); also www.genecard.org and <http://pirnabank.ibab.ac.in/> |
| Pseudogenes, retropseudogenes or other homologs? | D | Yes | Supporting Information S3: RT-qPCR methodology: controlled by the manufacturers (Qiagen, TIBMolBiol, Life technology); also www.genecard.org and <http://pirnabank.ibab.ac.in/> |
| Sequence alignment | D | Yes | Supporting Information S3: RT-qPCR methodology: controlled by the manufacturers (Qiagen, TIBMolBiol, Life technology); also www.genecard.org and <http://pirnabank.ibab.ac.in/> |
| Secondary structure analysis of amplicon | D | Yes | Putative secondary structure according to fRNAdb, see www.ncrna.org/frnadb |
| Location of each primer by exon or intron (if applicable) | **E** | Yes | Supporting Information S3: RT-qPCR methodology |
| What splice variants are targeted? | **E** | Yes | Supporting Information S3: RT-qPCR methodology |
| **qPCR oligonucleotides** |  |  |  |
| Primer sequences | **E** | Yes | Supporting Information S3: RT-qPCR methodology , determination of piRNAs; for miRNAs, the manufacturer does not provide this information |
| RTPrimerDB Identification Number | D | No |  |
| Probe sequences | D | Yes | Supporting Information S3: RT-qPCR methodology , determination of piRNAs; information not available for miRNAs |
| Location and identity of any modifications | **E** | Yes | Supporting Information S3: RT-qPCR methodology, determination of piRNAs; information not available for miRNAs |
| Manufacturer of oligonucleotides | D | Yes | Supporting Information S3: RT-qPCR methodology |
| Purification method | D | Yes | TIB Molbiol: probes HPLC, primers HPLC or GSF purification; other unknown |
| **qPCR protocol** |  |  |  |
| Complete reaction conditions | **E** | Yes | Main text: Materials and Methods; Supporting Information S3: RT-qPCR methodology |
| Reaction volume and amount of cDNA/DNA | **E** | Yes | Main text: Materials and Methods; Supporting Information S3: RT-qPCR methodology |
| Primer, (probe), Mg++ and dNTP concentrations | **E** | Yes | Main text: Materials and Methods; Supporting Information S3: RT-qPCR methodology |
| Polymerase identity and concentration | **E** | Yes | Main text: Materials and Methods; Supporting Information S3: RT-qPCR methodology |
| Buffer/kit identity and manufacturer | **E** | Yes | Main text: Materials and Methods; Supporting Information S3: RT-qPCR methodology |
| Exact chemical constitution of the buffer | D | No | The manufacturers does not provide this information |
| Additives (SYBR Green I, DMSO, etc.) | **E** | No |  |
| Manufacturer of plates/tubes and catalog number | D | Yes | Supporting Information S3: RT-qPCR methodology (Roche; Cat.No. 04729692001) |
| Complete thermocycling parameters | **E** | Yes | Main text: Materials and Methods; Supporting Information S3: RT-qPCR methodology |
| Reaction setup (manual/robotic) | D | Yes | Manual setup |
| Manufacturer of qPCR instrument | **E** | Yes | Main text: Materials and Methods: LightCycler 480 (Roche) |
| **qPCR VALIDATION** |  |  |  |
| Evidence of optimisation | D | Yes | For miRNAs: kits from Life Technology; for piRNAs:  Supporting Information S3: RT-qPCR methodology, determination of piRNAs, including Fig. S2A and S2B |
| Specificity (gel, sequence, melt, or digest) | **E** | Yes | Supporting Information S3: RT-qPCR methodology: Fig. S2A (melting curve) and S2B gel electrophoresis) |
| For SYBR Green I, Cq of the NTC | **E** | Yes | Supporting Information S3: RT-qPCR methodology, Figure S2B, lane C |
| Standard curves with slope and y-intercept | **E** | Yes | Supporting Information S3: RT-qPCR methodology, Table S4 |
| PCR efficiency calculated from slope | **E** | Yes |  |
| Confidence interval for PCR efficiency or standard error | D | Yes | Supporting Information S3: RT-qPCR methodology, Table S4 |
| r2 of standard curve | **E** | No | Not provided by the LC480 software |
| Linear dynamic range | **E** | Yes | Supporting Information S3: RT-qPCR methodology, Table S4 |
| Cq variation at lower limit | **E** | Yes | Supporting Information S3: RT-qPCR methodology, Table S4 |
| Confidence intervals throughout range | D | No |  |
| Evidence for limit of detection | **E** | Yes | Supporting Information S3: RT-qPCR methodology, Table S4: all samples with Cq values below 2 SD of the highest Cq of the generated standard curve |
| If multiplex, efficiency and LOD of each assay. | **E** | Not applicable |  |
| **DATA ANALYSIS** |  |  |  |
| qPCR analysis program (source, version) | **E** | Yes | Main text: Materials and Methods, quantitative RT-PCR; Supporting Information S3: RT-PCR methodology, Table S4 (LightCycler software, release 1.5.0 using the “second derivative maximum” method) |
| Cq method determination | **E** | Yes |  |
| Outlier identification and disposition | **E** | Not applicable |  |
| Results of NTCs | **E** | Yes | Supporting Information S3: RT-PCR methodology |
| Justification of number and choice of reference genes | **E** | Yes | Main test: Results, Differential expression of piRNAs; Supporting Information S3: RT-PCR methodology Reference to miR-28 and miR-106a according to ref. [10] |
| Description of normalisation method | **E** | Yes | Main test: Results: Differential expression of piRNAs, Legend to the Figure 1. Supporting Information S3: RT-PCR methodology Reference to miR-28 and miR-106a according to ref. [10] |
| Number and concordance of biological replicates | D | Yes | Main text: Table 1; Figure 1 |
| Number and stage (RT or qPCR) of technical replicates | **E** | Yes | Supporting Information S3: RT-PCR methodology, determinations of piRNAs; Table S5 |
| Repeatability (intra-assay variation) | **E** | Yes | Supporting Information S3: RT-PCR methodology and Table S5 |
| Reproducibility (inter-assay variation, %CV) | D | No | Supporting Information S3: RT-PCR methodology and Table S5 |
| Power analysis | D | Yes | Main text: Materials and Methods: Data analysis and statistics; Supporting Information S1 |
| Statistical methods for result significance | **E** | Yes | Main text: Materials and Methods: Data analysis and statistics; Supporting Information S1 |
| Software (source, version) | **E** | Yes | Main text: Materials and Methods: Data analysis and statistics; Supporting Information S1 |
| Cq or raw data submission using RDML | D | No |  |
| All essential information (E) must be submitted with the manuscript. Desirable information (D) should be submitted if available. | | | |

**In the following sections, the quantification of the reference genes miR-28 and miR106a, the three piRNAs, and analytical performance data for all the measurements are compiled.**

***Quantification of miR-28 and miR-106a as reference genes***

**The miRNAs miR-28 and miR-106a were used as normalizer according to previous results on suitable reference genes for miRNA expression in renal cell carcinomas [10]. TaqMan microRNA Reverse Transcription Kits of Life Technologies with details given in the following Supplemental Table S3 were used.**

**Supplemental Table S3.** TaqMan MicroRNA assays for the two reference genes miR-28 and miR-106a

TaqMan MicroRNA Assays (Life Technologies) were used for the measurement of the two mature miRNAs that were applied as normalizer genes for relative quantification of piRNAs. Assay names and assay IDs are taken from the nomenclature of the supplier Life Technologies. The miRBase accession no., miRBase ID, and the target sequences were taken from the miRBase database release 21 (Sanger Institute, Manchester, UK; [http://www.mirbase.org](http://www.mirbase.org/)).

| **Assay name**  **(Life Technologies)** | **Assay ID** | **miRBase Accession No.** | **miRBase ID**  **(v21)** | **Target Sequence** |
| --- | --- | --- | --- | --- |
| hsa-miR-28 | 000411 | MIMAT0000085 | hsa-miR-28-5p | AAGGAGCUCACAGUCUAUUGAG |
| hsa-miR-106a | 002169 | MIMAT0000103 | hsa-miR-106a-5p | AAAAGUGCUUACAGUGCAGGUAG |

The cDNA synthesis and real-time qPCR conditions were performed **according to the specified reaction conditions given by the manufacturer** (Life Technologies). Briefly, the cDNA synthesis was done in a 10 µl reaction volume (0.2 ml PCR SingleCap 8er-SoftStrips, Biozym Scientific GmbH, Oldenburg, Germany) and included: 10 nmol dNTP mix, 2.6 U RNase inhibitor, 33.5 U MultiScribe RT enzyme, 1 x RT Buffer, and 0.5x miRNA-specific stem-looped primers (Life Technologies). The transcription reaction was run in a thermal block cycler with heated lid (Biometra GmbH, Göttingen, Germany) and was carried out as follows: priming at 16°C for 30 min, transcription at 42°C for 30 min, and enzyme inactivation at 85°C for 5 min. All cDNA samples were stored at -20°C until PCR analysis. All real-time qPCR runs were performed on the LightCycler 480 Instrument (Roche Molecular Diagnostics, Mannheim, Germany) in white 96-well plates (Cat.No. 04729692001). One µl of miRNA-specific cDNA was used in a total PCR reaction volume of 10 µl per well. The reaction mix included also 5 µl TaqMan 2x Universal PCR Master Mix No AmpErase UNG, 1 µl gene-specific 20x TaqMan MicroRNA real-time PCR-Assay solution (Life Technologies), and 3.5 µl PCR grade water. The PCR run conditions were as follows: initial activation of Taq polymerase at 95°C for 10 min, 45 amplification cycles of 2-steps: denaturation at 95°C for 15 s, annealing/elongation at 60°C for 1 min with fluorescence acquisition and final cooling step at 40°C for 1 min. **All cDNA samples were measured in duplicates. Quantification cycles (**Cq values) were user independently calculated by the LightCycler software, release 1.5.0 based on the "second derivative maximum" method. **Data analysis was performed using the qBase PLUS software (Biogazelle,** Zwijnaarde**, Belgium); mean Cq values were used to calculate the normalized relative quantities of each piRNA with related to reference miRNAs. Further details that also apply for all RT-qPCR measurements were compiled in the previous S**upplemental Tables S2 (checklist of the MiQE guidelines) and the following Table S4 (characteristics of the PCR-standard curves), and S5 (precision data of PCR-measurements).

***Quantification of piRNAs***

**Different methods with customized assays were used to quantify the three piRNAs.** It was the primary intention to use the SYBR Green based **miScript PCR system from Qiagen** for the measurements of all three piRNAs. However, specificity controls of the piRNA RT-qPCR products (melting curve analyses and electrophoretic characterization of the PCR products) showed, except for piR- **piR-57125,** insufficiently specific products for **piR-30924 and piR-38756 measurements despite the application of** several designed primers and numerous optimisation experiments. Thus, TaqMan assays were used as alternatives. In all cases, the expression of piRNAs and also miRNAs were quantified by using standard curves. **The quantification of piR-30924 and piR-38756 generally followed the RT-qPCR principles of TaqMan microRNA Assays from Life Technologies using RT-stem-loop primers and qPCR product detection with specific hydrolysis probes (TaqMan probes).**

**Determination of piR-30924.** **The primer and probes for the determination of piR-30924 were designed by TIB MOLBIOL GmbH (Berlin, Germany) and had following sequences (5'-3’ direction): the stem-loop RT-primer:** gCCTCTCAAgCTgACgAATTATgAgAggCgACgCTTT, the PCR primers: forward, ACTCgCTTCCAgAgAggg; reverse, gCCTCTCAAgCTgACgAAT. The TaqMan probe is labeled with Fluorescein (FAM) at the 5’-end and with BlackBarry Quencher (BBQ) at the 3’-end. The hydrolysis probe sequence is: 6FAM-AggCgACgCTTTCCAAggC-BBQ.

The RT reaction conditions corresponded to the standard TaqMan miRNA transcription conditions as described in the section of miRNA measurements. Different RT and PCR optimization tests were necessary and resulted in a RT-stem-loop primer final concentration of 60 nM at a total RNA input of 40 ng per well. The qPCR was an asymmetric PCR with a final concentration of the forward primer of 1.5 µM and one of the reverse primer of 0.7 µM. Both steps were followed in adaptation to protocols described by Chen et al. [9] and Kramer et al. [11]. The final TaqMan probe concentration was 0.2 µM. All other PCR mix components and run conditions were exactly the same as described for miRNA measurements. F**urther general details for all RT-qPCR measurements were compiled in the S**upplemental Tables S2, 4, 5.

The product size was checked by agarose gel electrophoresis (see the following Supplemental Fig. S2, lane E). The detected fragment of ~67 bp corresponded to the piR-30924 specific primer design with an expected product size of 68 bp.

**Determination of piR-57125.** **A miScript Primer Assay for piR-57125 was custom-designed from Qiagen. The forward primer had the sequence of 5’-GTCGTGGTTGTAGTCCGTGCGA-3’.**

The RT reaction was performed in 10 µl final volume and included: 2 µl 5x miScript HiFlex Buffer, 1 µl 10x miScript Nucleics Mix, 4 µl RNase-free water, and 2 µl RNA (total of 3 ng RNA per tube). The transcription reaction was run in a thermal block cycler with heated lid (Biometra) at 37°C for 60 min and was finished with the inactivation of miScript Transcriptase Mix at 95°C for 5 min. All miScript cDNA samples were stored at -20°C until PCR analysis.

One µl of miScript cDNA was used in a total PCR reaction volume of 12.5 µl per well. The reaction mix included also 6.25 µl 2x QuantiTect SYBR Green PCR Master Mix, 1.25 µl 10x miScript Universal Primer (reverse), 1.25 µl 10x pi-59 specific miScript Primer Assay (forward), and 2.75 µl RNase-free water. The PCR run conditions were as follows: initial activation step at 95°C for 15 min, 40 amplification cycles of 3-steps: denaturation at 94°C for 15 s, annealing at 55°C for 30 s, extension at 72°C for 30 s with fluorescence acquisition and one melting curve analysis step with at first denaturation at 95°C for 5 s and a second product melting step from 65°C up to 95°C with a ramping rate of 011°C/s and continuous fluorescence acquisition. The PCR ended with a cooling step at 40°C for 1 min. F**urther general details for all RT-qPCR measurements were compiled in the S**upplemental Tables S2, 4, 5).

The qPCR primer specificity was confirmed by melting curve analysis with a resulting single sharp melting peak at 78°C (see the Supplemental Fig. S2A) and by agarose gel electrophoresis with detection of a single fragment of 86 bp size (see the Supplemental Figure S2B, lane B; fragment size was not given by Qiagen).

**Determination of piR-38756.** **A Custom TaqMan small RNA Assay (Life Technologies) was used for the determination of piR-38756. The design strategy of this assay is equal to the general TaqMan microRNA Assays as described above. The primer sequences for RT reaction and for the subsequent qPCR were not disclosed by the manufacturer, but the name of the designed assay was given (Assay-ID: CSXOZUJ). The reaction conditions for the reverse transcription and qPCR corresponded to the reaction conditions used for the quantification of miRNAs as described above. Further general details for RT-qPCR measurements were compiled in the S**upplemental Tables S2, 4, 5.

**The agarose gel electrophoresis (see the following Supplemental Fig. S2, lane C) shows a single PCR fragment band with a size of ~59 bp. The correct size was not disclosed by the manufacturer. Information regarding the standard curve and precision data are given in the Supplemental Tables S2 and S3.**

***Performance data of the RT-qPCR analyses***

**Supplemental Figure S2. Controls of the specificity of the RT-PCR products of the piRNA measurements**

**A). Melting curve analysis on the LightCycler 480 of piR-57125 specific RT-qPCR product.**

A single melting peak at 78°C confirms the reliability of primer design and PCR run conditions for quantification of piR-57125


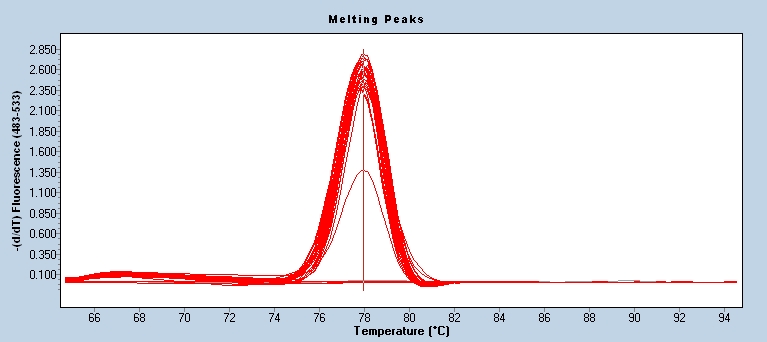


**B). Agarose gel electrophoresis of the piRNA RT-qPCR products**

**A B C D E**

| **Lane A**  **piR-57125** | **Lane B**  **piR-57125; NTC** | **Lane C**  **piR-38756** | **Lane D**  **Marker VIII** | **Lane E**  **piR-30924** |
| --- | --- | --- | --- | --- |
| **86** |  | **59** | **320** | **67** |
|  |  |  | **242** |  |
|  |  |  | **190** |  |
|  |  |  | **147** |  |
|  |  |  | **124** |  |
|  |  |  | **110** |  |
|  |  |  | **67** |  |
|  |  |  | **30 (19-37)** |  |

Fragment sizes given in bp.

Agarose gel electrophoresis conditions: 3% agarose gel (Biozyme Small DNA low melt Agarose); TBE-running and gel buffer both including ethidium bromide (0.5 µg/ml); Gel Loading Solution (Sigma, Traufkirchen, Germany, Cat. No. G2526). Run conditions: 1 h, 33 mA; 120V; Biometra Agarose Mini chamber; gel size: 7x10 cm; 0.5 cm thick. Per lane: 8 µl amplicon + 2 µl Gel Loading Solution. The amplicon from piR-57125 RT-qPCR was 1:10 pre diluted with TBE-buffer w/o ethidium bromide. The gel bands were documented with the Multi Imager System Fusion FX7 (Vilber Lourmat GmbH, Eberhardzell, Germany). The calculation of fragment size was performed with the Fusion Capt Advanced Software using the DNA Molecular Weight Marker VIII (Roche Diagnostics, Cat.No. 11336045001). Only the eight shortest marker of the 17 markers were included in the size calculation of piR-PCR products.

**Supplemental Table S4.** Characteristics of the standard curves of qPCR analyses

Standard curves were generated either from diluted cDNAs or from diluted amplicons. Cq values were calculated by the LightCycler Software Version 1.5.0 using the "second derivative maximum" method. The efficiency, the slope, intercept, and error of the regression line as well as the so-called dynamic range and the Cq variation at the lower limit (the endpoint of the dynamic range) were calculated by the LightCycler 480 software.

| **Gene** | **PCR efficiency^1^** | **Slope** | **y-Intercept** | **Error^2^** | **Dynamic range^3^** | **Cq variation at lower limit (SD)^4^** |
| --- | --- | --- | --- | --- | --- | --- |
| **miR-28**  **miR-106a** | 1.934 | -3.491 | 23.74 | 0.0324 | 23.41-34.96 | 0.20 |
| **piR-30924** | 1.915 | -3.543 | 20.54 | 0.0239 | 20.73-35.10 | 0.65 |
| **piR-57125** | 1.941 | -3.473 | 8.696 | 0.0048 | 8.96-27.96 | 0.25 |
| **piR-38756** | 1.806 | -3.894 | 17.73 | 0.00251 | 17.54-36.16 | 0.66 |

^1^The PCR-efficiency is calculated by the LightCycler480 software after the formula: Efficiency=10^-1/slope^

^2^According to the LightCycler 480 operator’s manual, the error value is the mean squared error of the single data points fit to the regression line.

^3^Dynamic range represents the range of mean Cq values between the highest and the lowest Cq values of the generated standard curve.

^4^Cq variation given as SD at the lower limit, which is defined as the endpoint of the dynamic range.

**Supplemental Table S5.** Intra-run and inter-run precision data of qPCR measurements

| **Target gene** | **Intra-run precision** | | | **Inter-run precision** | | |
| --- | --- | --- | --- | --- | --- | --- |
|  | Repeats | %RSD (Cq)^1^ | %RSD (AU)^2^ | Repeats | %RSD (Cq)^1^ | %RSD (AU)^2^ |
| miR-28 | 8 | 0.40 | 7.3 | 5 | 0.24 | 4.2 |
| miR-106a | 8 | 0.40 | 6.6 | 5 | 0.28 | 4.4 |
| piR-30924 | 6 | 0.15 | 2.8 | 5 | 0.65 | 13.1 |
| piR-57125 | 4 | 0.32 | 2.4 | 6 | 1.15 | 8.8 |
| piR-38756 | 8 | 0.46 | 8.40 | 7 | 0.42 | 6.68 |

^1^%RSD (Cq) corresponds to the percent relative standard deviation calculated on the basis of the Cq values.

^2^%RSD (AU) corresponds to the percent relative standard deviation calculated on the basis of the normalized relative quantities as arbitrary units (AU).

**SUPPORTING INFORMATION S4: Additional figures**

**Supplemental Fig. S3.** Expression of piRNAs in association to tumour stage and histological classification according to Fuhrman grading

Values are given as geometric means with 95% confidence intervals. Differences between samples from normal adjacent tissue (blank columns), pT1+2 or Fuhrman grade 1+2 (gray columns), and pT3+4 or Fuhrman grade 3+4 (black columns) were tested by the Student's t test with log-transformed data.

**References in the Supporting Information**

References

1. Wotschofsky Z, Busch J, Jung M, et al. Diagnostic and prognostic potential of differentially expressed miRNAs between metastatic and non-metastatic renal cell carcinoma at the time of nephrectomy. Clin Chim Acta. 2013;416:5-10.

2. Veeratterapillay R, Simren R, El-Sherif A, Johnson MI, Soomro N, Heer R. Accuracy of the revised 2010 TNM classification in predicting the prognosis of patients treated for renal cell cancer in the north east of England. J Clin Pathol. 2012;65:367-71.

3. Sai Lakshmi S, Agrawal S. piRNABank: a web resource on classified and clustered Piwi-interacting RNAs. Nucleic Acids Res. 2008;36:D173-D177.

4. Zhang P, Si X, Skogerbo G, et al. piRBase: a web resource assisting piRNA functional study. Database (Oxford). 2014;2014:bau110.

5. Sarkar A, Maji RK, Saha S, Ghosh Z. piRNAQuest: searching the piRNAome for silencers. BMC Genomics. 2014;15:555.

6. Belinky F, Bahir I, Stelzer G, et al. Non-redundant compendium of human ncRNA genes in GeneCards. Bioinformatics. 2013;29:255-61.

7. Rosenkranz D, Zischler H. proTRAC - a software for probabilistic piRNA cluster detection, visualization and analysis. BMC Bioinformatics. 2012;13:5.

8. Bustin SA, Benes V, Garson JA, et al. The MIQE guidelines: minimum information for publication of quantitative real-time PCR experiments. Clin Chem. 2009;55:611-22.

9. Chen C, Ridzon DA, Broomer AJ, et al. Real-time quantification of microRNAs by stem-loop RT-PCR. Nucleic Acids Res. 2005;33:e179.

10. Wotschofsky Z, Meyer HA, Jung M, et al. Reference genes for the relative quantification of microRNAs in renal cell carcinomas and their metastases. Anal Biochem. 2011;417:233-41.

11. Kramer MF. Stem-loop RT-qPCR for miRNAs. Curr Protoc Mol Biol. 2011;95:15.10.1-15.10.15.
